# Assessment of protein set coherence using functional annotations

**DOI:** 10.1186/1471-2105-9-444

**Published:** 2008-10-20

**Authors:** Monica Chagoyen, Jose M Carazo, Alberto Pascual-Montano

**Affiliations:** 1 Centro Nacional de Biotecnología – CSIC, Madrid, Spain; 2 Dpto. Arquitectura de Computadores y Automática, Universidad Complutense Madrid, Madrid, Spain

## Abstract

**Background:**

Analysis of large-scale experimental datasets frequently produces one or more sets of proteins that are subsequently mined for functional interpretation and validation. To this end, a number of computational methods have been devised that rely on the analysis of functional annotations. Although current methods provide valuable information (e.g. significantly enriched annotations, pairwise functional similarities), they do not specifically measure the degree of homogeneity of a protein set.

**Results:**

In this work we present a method that scores the degree of functional homogeneity, or coherence, of a set of proteins on the basis of the global similarity of their functional annotations. The method uses statistical hypothesis testing to assess the significance of the set in the context of the functional space of a reference set. As such, it can be used as a first step in the validation of sets expected to be homogeneous prior to further functional interpretation.

**Conclusion:**

We evaluate our method by analysing known biologically relevant sets as well as random ones. The known relevant sets comprise macromolecular complexes, cellular components and pathways described for *Saccharomyces cerevisiae*, which are mostly significantly coherent. Finally, we illustrate the usefulness of our approach for validating 'functional modules' obtained from computational analysis of protein-protein interaction networks. Matlab code and supplementary data are available at

## Background

An increasing number of functional data are available at different genome databases and resources spanning all biological levels. Functional information is usually provided as annotations associated with gene products using functional terms from controlled vocabularies and ontologies [1]. This information is being exploited to perform 'functional computations' in quite different contexts and applications. A first classification of these functional methods distinguishes between predictive and descriptive approaches.

Predictive approaches are intended to infer new functional annotations for a gene product or a set of them from available data (some recent reviews can be found [2-4]). Most methods use implicit functional information from experimental data (e.g. sequences, gene expression data, protein-protein interactions or phylogenetic profiles) while some approaches rely only on explicit functional information such as existing annotations [5-7] or a combination of annotations and literature references [8].

In contrast, descriptive approaches are intended to perform functional validation and interpretation of experimental results. The objective of these methods is to compare new experimental data with the current state of knowledge as stored in curated databases. In this way, experimental data can be validated and new insights can be highlighted from the analysis. Among descriptive methods, a distinction can be made between those that perform functional analysis of a protein set and those that perform pairwise functional analysis.

Given a set of proteins obtained from experimental or computational analysis, currently available methods are able to extract those functional annotations that best describe that protein set [9-11] or to classify it into subsets using functional annotations [12-15]. Nevertheless, the most widely-used functional methods for analyzing protein sets are those described as annotation 'enrichment'. These methods are used to find functional terms that are statistically significant in a protein set given a reference set (typically a whole organism or the genes spotted in a DNA microarray). A large variety of tools are available to perform such analyses (see a recent review [16] or the Gene Ontology (GO) web site ). Those tools first retrieve all annotations of a protein set of interest from a functional scheme. The number of proteins annotated with each functional term is then counted in both the input and reference sets. Finally, a statistical test (e.g. χ^2^, binomial, hypergeometric or Fisher's exact test) is applied to measure the significance of each functional term, and this is subsequently adjusted for multiple testing. The result of this type of analysis is therefore a list of functional terms with their corresponding p-values. Those terms with p-values indicating statistical significance are considered representative and therefore give information about the 'enriched' functions in the protein set. Although some methods have been developed to obtain enriched co-annotations (e.g. [17]), most tools analyze functional terms independently, thus providing a view of the local significant functions of a protein set.

In addition, several studies have been reported that aim to establish a similarity score for a pair of proteins, accounting for the resemblance of their functional annotations. To this end, several similarity measurements have been described [13,15,18-23], each following different, though in many cases related, approaches. Pairwise protein similarities can be computed through a combination of functional term-term similarities (as in [21]) or by measuring global protein-protein functional similarity directly (as in [13]). These measurements can be applied to any controlled vocabulary scheme, although most of them exploit the hierarchical nature of functional ontologies such as Gene Ontology [24] and the MIPS Functional Catalogue (FunCat) [25].

Although these methods provide valuable information, they do not specifically address the issue of functional homogeneity, i.e. whether a set of proteins participates in related cellular processes, performs similar molecular activities, confers similar phenotypes, etc. An experimental set of proteins is usually grouped on the basis of shared experimental features (gene expression profiles, interaction partners, etc), and it is expected that such a set can be distinguished from a random set when considering a particular functional aspect. Therefore, a method that measures the degree of overall functional homogeneity of a protein set would be useful for validating experimentally or computationally derived sets, highlighting those that merit further investigation. For example, when protein-protein interaction networks are analyzed to discover functional modules, protein clusters could first be filtered on the basis of functional homogeneity, avoiding any additional functional interpretation for those heterogeneous cases.

To this end we propose a new descriptive method, based on functional annotations, that evaluates the statistical significance of the overall homogeneity of a protein set. Given a set of proteins, we first compute its degree of homogeneity (in terms of a functional *coherence score*) accounting for the global similarity of their functional profiles. This coherence score is computed using a previously-reported global pairwise functional similarity measure. Then we assess whether this score is statistically significant given a reference set (usually a complete organism, or the set of genes present in the experimental setting). This significance is measured in terms of the number of proteins in the reference that are also similar to the set at its particular coherence level. Note that a very homogeneous protein set (with a high coherence score) will not be statistically significant in the context of a reference set if it contains only a few proteins of the reference that are functionally related. On the other hand, a relatively homogeneous set (with a lower coherence score) might be significant if it contains a sufficient number of functionally related proteins of the reference.

To the best of our knowledge no previous method relying on functional annotations has addressed this task specifically. Nevertheless, previous studies have sought to evaluate the overall functional coherence of a set of proteins using literature analysis [26,27]. In these methods a coherence score is assigned to a group of proteins from the perspective of the relevant published literature. The literature is known to report information that is both related and complementary to functional annotations [28]. It is therefore expected that the overall functional coherence of a protein set could also be computed from functional annotations. Nevertheless, it is not obvious how to compute that overall functional coherence from the output of current enrichment analysis tools. As noted by Zheng and Lu [27], standard enrichment methods present some drawbacks, including: (i) they ignore the relationships among GO terms; (ii) when multiple GO terms are 'enriched' within a protein group, it is difficult to derive a quantitative metric that gives and overall reflection of the functional relationships of the proteins or their statistical significance evaluations. In the present work, we have addressed these limitations by providing a complementary descriptive method that (i) considers relationships among functional terms, both hierarchical and arising from co-annotation, (ii) measures the overall functional homogeneity of a protein set and its statistical significance.

## Methods

### Protein representation

A protein is represented as an *n*-dimensional vector, each dimension corresponding to one of the *n *functional annotations of the reference set (in this work, the complete genome). Therefore, each functional term will correspond to a coordinate of the vector space representation. In the case of hierarchical functional schemes (e.g. Gene Ontology and MIPS FunCat) this representation is constructed by assigning 1 to each functional term annotated to a gene product and to its corresponding ancestor terms in the hierarchy. The remaining vector coordinates are equal to 0.

To account for the specificity and generality of functional terms, a weighting scheme is applied to this vector representation using the information content of each term. The information content (*IC*) of a term is inversely related to its probability of annotation in the reference set Pr(*t*). The weight is formally calculated as:

(1) *w*_*IC*_(*t*) = -*ln*(Pr(*t*)) = -ln(#*genes*_*t*_/*m*)

where Pr(*t*) is the probability of annotation of a term *t*, estimated as the number of gene products associated with *t *(#genes_t_) divided by the total number of protein-term associations (*m*) in a reference set R. Note that the total number of gene products associated with *t *is the sum of those directly annotated with *t *and those annotated with any of its descendants in the functional hierarchy.

### Similarity measure

The similarity between two proteins *p*_*i *_and *p*_*j *_is computed using the cosine similarity of their corresponding functional representations, as in [29]:

(2) $$sim(p_{i},p_{j})=\frac{p_{i}•p_{j}}{\left|p_{i}\right|\left|p_{j}\right|}=\frac{p_{i}•p_{j}}{\sqrt{p_{i}•p_{i}}\sqrt{p_{j}•p_{j}}}$$

where *p*_*i *_• *p*_*j *_is the dot product between the two vectors *p*_*i *_and *p*_*j*_.

The similarity between a protein *p *and a set of proteins P is defined as the average pairwise similarity of the protein *p *to each protein in the set:

(3) $$sim(p,\text{P})=\frac{\sum_{p_{i}\in \text{P}}sim(p,p_{i})}{\left|\text{P}\right|}$$

where |P| denotes the cardinality of the set, i.e. the number of distinct elements it contains.

### Coherence score

The coherence score of a set of proteins of interest, S, is defined in this work as the average functional similarity of its distinct protein pairs:

(4) $$score(\text{S})=\frac{\sum_{i=1}^{\left|\text{S}\right|}\sum_{j=i+1}^{\left|\text{S}\right|}sim(p_{i},p_{j})}{\left|\text{S}\right|(\left|\text{S}\right|-1)/2}$$

Therefore, the coherence score will range from 0 (no coherence) to 1 (full coherence, corresponding to exactly the same functional annotations for all proteins in S).

### Statistical significance of the coherence score

To assess the significance of the coherence score calculated for a set S in the context of a reference set R, we take into account the proteins in R that are functionally related to S. The definition of functional-relatedness is somewhat arbitrary. Therefore, for evaluation purposes, we use three different criteria to decide whether a protein is functionally related to the set S. In turn, these three criteria define three neighbourhoods in the *n*-dimensional functional space. Therefore, each criterion is established for a set in the context of a reference and for the particular coherence score obtained for the set as computed in equation (4). These three criteria are as follows.

• The first criterion defines proteins to be functionally related to S if their similarity to the set, as defined in equation (3), is greater than or equal to the coherence score of the set. This establishes a neighbourhood around the most homogeneous proteins of the set. Proteins in S fulfilling this criterion are defined as the 'core' of S, denoted as C(S). Thus, according to the first criterion, a protein *p *∈ R is functionally related to S if sim (*p*, S) ≥ score(S).

• The second criterion defines proteins to be functionally related to S if their similarity to at least one protein in C(S), as defined in equation (2), is greater than or equal to the coherence score of the set. This second neighbourhood can be described as open to the core of the set (as it captures proteins similar to one protein in the core). Thus, according to the second criterion, a protein *p *∈ R is functionally related to S if ∃*p*_*i *_∈ C(S), sim(*p*, *p*_*i*_) ≥ score(S).

• The third criterion defines proteins to be functionally related to S if their similarity to at least one protein in S, as defined in equation (2), is greater than or equal to the coherence score of the set. This third neighbourhood is open to the set (as it captures all proteins similar to one protein in the set). Thus, according to the third criterion, a protein *p *∈ R is functionally related to S if ∃*p*_*j*_∈ S, sim(*p*, *p*_*j*_) ≥ score(S)

**Figure 1:**
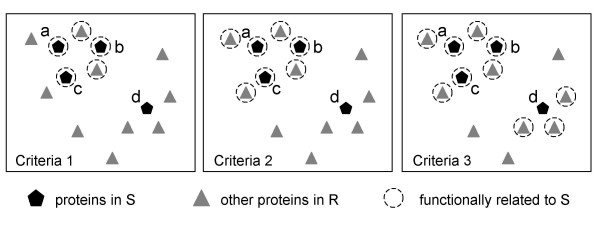
**Functional relationships**. Illustration of the three criteria established to assess the significance of the coherence score of a protein set (S) in the context of a reference set (R). Proteins in S are represented as pentagons (namely proteins a, b, c and d), and the remaining proteins in R as triangles. Dashed circles surround proteins functionally related to S according to the definitions provided in the Methods section. Proteins a, b and c form the 'core' of the set S.

The numbers of proteins in S and in R that fulfil each criterion are counted and denoted *s *and *r *respectively (see Figure 1). Each criterion can be also interpreted as attaching a binary variable ('functionally related to S') to each protein in the reference set: those that fulfil the criterion are tagged as 'functionally related to S', and those that do not as 'non-functionally related'. The proteins can also be described by a second binary variable ('S membership'). In this way, a 2 × 2 contingency table is built. The independence of these two variables ('functionally related to S' and 'S membership') can be assessed by computing the cumulative hypergeometric distribution, where *p-value *is defined as:

(5) $$p-value=\sum_{i=s}^{\left|\text{S}\right|}\frac{\left(\begin{array}{c} r\\ i \end{array}\right)\left(\begin{array}{c} \left|\text{R}\right|-r\\ \left|\text{S}\right|-i \end{array}\right)}{\left(\begin{array}{c} \left|\text{R}\right|\\ \left|\text{S}\right| \end{array}\right)}$$

Given a reference set R with *r *elements 'functionally related to S', *p-value *gives the probability of drawing *s *or more elements 'functionally related to S' when |S| elements are selected from R at random. In this work, |S| is the cardinality of the protein set to be analyzed, and |R| is the total number of gene products in the genome taken as reference. We obtain a *p-value *for each of the criteria described above (pv1, pv2 and pv3 respectively).

In summary, the coherence score of a protein set provides a global measure of the functional homogeneity of its proteins. Meanwhile, the significance measures we propose (pv1, pv2, pv3) account for the probability of obtaining a set from a reference with a given number of proteins functionally related to that set, just by chance. Note that the definitions of the three criteria for functional relatedness depend on the coherence score of the set. In this sense, the greater the coherence score, the fewer proteins in the reference will be found to be functionally related. Nevertheless, a particular set with a high coherence score might not be significantly coherent given the reference if it contains only a few of the proteins in the reference that are functionally related to the set (the exact number of proteins to be significant depends on both the size of the set and the number of similar proteins in the reference). Meanwhile, a set with a relatively low coherence score can be significantly coherent with respect to a reference if it contains a certain number of proteins of the reference that are functionally related at that coherence level.

## Results

We have assessed the validity of our method by performing several analyses. First, we evaluate the method by comparing the results obtained from the analysis of protein sets known to be homogeneous to those obtained from randomly created sets. Secondly, we analyze its robustness in terms of the functional similarity used, the completeness of functional annotation of the organism and the inclusion or exclusion of annotations obtained by automatic methods. Finally, we demonstrate the usefulness of our approach for a particular application: the validation of functional modules obtained from the analysis of protein-protein interaction networks.

### Evaluation

To assess the validity of our method for characterizing the functional coherence of a set of proteins, as well as its significance, we analyzed both positive and random sets in the context of one of the most complete and expert-validated annotated genomes: *Saccharomyces cerevisiae*. In this scenario, our positive sets (those that are expected to be functionally homogeneous) correspond to macromolecular complexes, cellular components and proteins participating in the same pathway. As proteins in a complex or component act co-ordinately, participating in one or more cellular processes, these protein sets are expected to be significantly coherent from the biological process point of view. The same is expected in the case of proteins in the same pathway. Therefore, we restrict our analysis to GO 'biological process' terms (Gene Ontology annotation release 2007–12).

**Figure 2:**
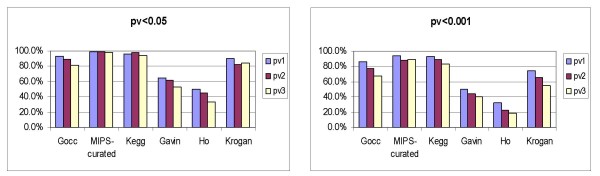
**Significant protein sets (*S. cerevisiae*)**. The percentages of significant (pv < 0.05) and highly significant (pv < 0.001) *S. cerevisiae *protein sets from curated databases (GO cellular components, MIPS complexes, and Kegg pathways), and complexes from systematic studies in the MIPS complex database (Gavin *et al*. [32], Ho *et al*. [33] and Krogan *et al*. [34]).

Specifically, positive sets from *S. cerevisiae *were compiled from (i) the Gene Ontology cellular component ontology (release 2007–12), (ii) MIPS complex catalogue (release 18-05-2006) available from the CYGD [30] and (iii) Kegg Pathways [31] (downloaded 17-12-2007). For each protein set we computed the coherence score in terms of GO 'biological process' annotations as well as corresponding significance measures in the context of the whole genome. The results of the analysis are available at the project web site. The proportion of statistically significant (p-value < 0.05) and statistically highly significant (p-value < 0.001) sets according to the three criteria proposed are shown in Figure 2.

#### Kegg pathways

98 protein sets containing at least two proteins annotated with a 'biological process' term were compiled from the Kegg pathways of *S. cerevisiae*. The coherence scores of these sets are in the range of 0.06–1, with set sizes between 2 and 147 proteins. Only 4 pathways are not significantly coherent (pv1 > 0.05), namely 'Limonene and pinene degradation', 'Lipoic acid metabolism', 'Tryptophan metabolism' and 'Alkaloid biosynthesis II'.

#### GO cellular components

For each GO cellular component (GOcc) term we created a protein set comprising the gene products annotated in the *S. cerevisiae *genome. Both direct and hierarchical associations were considered, so a set comprises all the gene products directly annotated with a GOcc term as well as those annotated with its descendants in the GO structure. As some GOcc terms comprised exactly the same set of proteins, we analyzed only distinct sets. Of the 552 distinct protein sets, we analyzed 503 that contained at least two proteins annotated in the 'biological process' category. The coherence scores range from 0.01 (less coherent) to 1 (most coherent), with set sizes in the range 2 to 4682 gene products (corresponding to GO:0005623 'cell'). A simple estimator of 'random' similarity is the average similarity between all possible protein pairs (mean pairwise similarity). Sets that are not significantly coherent according to the three criteria established, with coherence scores above these mean pairwise similarities in the *S. cerevisiae *genome, are shown in Table 1.

**Table 1:** Non-significant cellular components

**GO**	**Cellular component**	**Size**	**Score**	**p-value**	**s**	**r**
				1.08E-01	2	752
GO:0005641	nuclear envelope lumen	4	0.22	4.32E-01	2	1754
				6.22E-01	2	2314
				2.36E-01	2	944
GO:0031588	AMP-activated protein kinase complex	5	0.18	1.08E-01	4	2148
				2.71E-01	4	2828
				2.07E-01	2	1480
GO:0000306	extrinsic to vacuolar membrane	3	0.17	5.10E-01	2	2560
				6.47E-01	2	3027
				4.35E-01	3	1970
GO:0031314	extrinsic to mitochondrial inner membrane	6	0.14	5.13E-01	4	2955
				6.10E-01	4	3192
				6.33E-01	2	1934
GO:0031902	late endosome membrane	5	0.14	3.25E-01	4	2996
				7.84E-01	4	4159
				1.60E-01	3	1045
GO:0005775	vacuolar lumen	7	0.13	2.29E-01	4	1855
				2.40E-01	6	3301
				2.05E-01	4	1105
GO:0031307	integral to mitochondrial outer membrane	11	0.12	7.20E-01	5	2513
				8.66E-01	9	4449
				3.30E-01	7	2223
GO:0031312	extrinsic to organelle membrane	13	0.12	6.42E-02	12	3540
				4.45E-01	12	4352
				3.41E-01	3	1739
GO:0009898	internal side of plasma membrane	6	0.12	4.98E-01	5	3709
				8.66E-01	5	4496

GO cellular components with coherence scores above mean pairwise similarity in reference set, which are not significant (p-value > 0.05) according to the three criteria. Size of reference set |R| is 5051 gene products.

#### MIPS complexes

The catalogue of MIPS complexes comprises both curated data and the results of systematic analyses of protein complexes based solely on high-throughput methods [32-34]. We have analyzed those complexes separately (see Figure 2). Two hundred and seventeen protein sets corresponding to expert-annotated complexes contained at least two proteins with 'biological process' annotations. Their coherence scores range from 0.07 to 1, with set sizes in the range 2 to 81 proteins. Only two of these were not significant according to pv1: 'Mitochondrial processing complexes' (440.20) and 'DNA helicases' (410.40.40). The data from systematic analyses included 224 sets obtained by Gavin *et al*. [32], 532 by Ho *et al*. [33] and 62 by Krogan *et al*. [34].

#### Random sets

In order to ensure that our method does not provide significant sets by chance, we analyzed various randomly created sets of different sizes. Out of a total of 100,000 random sets, with a uniform size distribution from 2 to 200 proteins at 2-protein intervals (similar to the sizes of most positive sets), 4455 were found to be statistically significant (p-value < 0.05) according to pv1, 4379 according to pv2 and 682 according to pv3. These figures imply an FDR at or below a p-value of 0.05 using pv1, 0.045 using pv2 and a lower 0.0068 using pv3. The numbers of highly significant sets (p-value < 0.001) drop to 115 (pv1), 104 (pv2) and 20 (pv3) (with corresponding FDR at or below a p-value of 0.001 of 0.0015 using pv1, 0.0010 using pv2 and a lower 0.0002 using pv3). Additional file 1 shows the coherence scores and p-values (pv1, pv2 and pv3) of random sets plotted against size. As expected, the coherence scores of larger random sets tend towards the mean pairwise similarity of the whole genome (0.115).

As shown in Figure 2, expert-annotated datasets (GOcc annotations, curated MIPS complexes and Kegg pathways) are mostly significant (e.g. 94–99% with pv1 < 0.05). Nevertheless, they exhibit a wide range of coherence scores, in some cases even less than that expected by chance. This means that most sets corresponding to known macromolecular complexes, cellular components and pathways are significant in the context of the global functional landscape of *S. cerevisiae*, though some of them are quite heterogeneous. On the other hand, the proportion of significantly coherent sets corresponding to complexes derived from high-throughput methods stored in the MIPS catalogue [32-34] is lower than the expert-annotated datasets according to the three criteria (see Figure 2). Furthermore, the results of the analysis of random sets confirm that the probability of obtaining significant and highly significant coherence scores in such sets is very low.

As most expert-annotated data on known biologically meaningful sets are statistically significant, while the probability of obtaining significant sets just by chance is low, the measures proposed in this work seem to be valuable criteria for assessing the significance of the functional coherence of a protein set. Therefore, this significance can be used as a means of validating new experimental or hypothetical functional modules (e.g. co-expressed genes, protein-protein interaction clusters).

### Robustness

To evaluate the extent to which the statistical significance of the coherence score depends on various conditions such as functional similarity and completeness of annotation, we conducted the following experiments.

#### Functional similarity

The coherence score and the neighbourhoods constructed depend on a particular definition of global functional similarity. Therefore, we wanted to test the effect of choosing a different functional similarity measure. For that purpose we used Jaccard similarity on a set representation of gene products. The similarity between two gene products A and B is computed as the ratio of the number of common terms to the number of terms in A and B, as defined in equation (6), with no weights accounting for specificity/generality of terms. The number of significant sets among the expert-annotated datasets was, in general, slightly lower than the results obtained using cosine similarity (see Table 2 for details).

**Table 2:** Significant sets, Jaccard similarity

**Dataset**	**Sets**		**p-value < 0.05**	**p-value < 0.001**
GOcc	503	pv1	460 (-10, -1.99%)	425 (-9, -1.79%)
		pv2	440 (-9, -1.79%)	384 (-8, -1.59%)
		pv3	381 (-19, -3.78%)	320 (-18, -3.58%)
MIPS-curated	217	pv1	214 (-1, -0.46%)	201 (-3, -1.38%)
		pv2	212 (-3, -1.38%)	182 (-9, -4.15%)
		pv3	207 (-6, -2.76%)	180 (-13, -5.99%)
Kegg	98	pv1	94 (0, 0.00%)	91 (0, 0.00%)
		pv2	92 (-4, -4.08%)	88 (-1, -1.02%)
		pv3	92 (0, 0.00%)	83 (+1, +1.02%)

Number of significant and highly significant sets within expert-annotated data (GO cellular components, MIPS-curated complexes and Kegg pathways) analyzed using Jaccard similarity. Increase/decrease from analysis using cosine similarity is shown in parenthesis.

(6) $$sim_{Jaccard}(A,B)=\frac{\left|A\cap B\right|}{\left|A\cup B\right|}$$

#### Annotation

**Figure 3:**
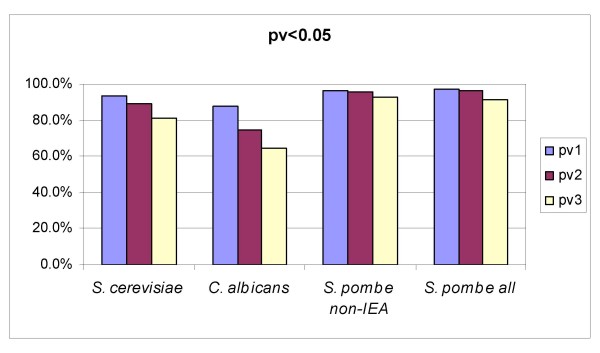
**Significant protein sets**. The percentage of significant protein sets (pv < 0.05) corresponding to GO cellular components from different organisms: *S. cerevisiae*, *C. albicans*, *S. pombe *(only non-IEA associations) and *S. pombe *(all associations).

Current functional annotation schemes are not complete and therefore, in our particular analysis, a number of biological processes might still not be described in detail. Also, the proportion of gene products annotated depends on both the level of functional characterization of an organism and the maturity of genome annotation projects. In order to verify whether this has an impact on the significance of coherent sets, we analyzed the GO cellular components from *Candida albicans*, an incipient project in which only an estimated 15.5% of the gene products have so far been annotated (see annotation statistics in Table 3). We can guess the degree of completeness of an organism as the percentage of gene products annotated and the number of distinct terms used. Assuming *S. cerevisiae *and *C. albicans *to be comparable in terms of biological complexity, we see that the current functional space of *C. albicans *is more incomplete in terms of biological processes than that of *S. cerevisiae *(nearly half the terms and fewer annotations per term). As expected from these data, the proportion of statistically significant coherent sets among cellular components in *C. albicans *is lower than in *S. cerevisiae *(see Figure 3). Nevertheless, pv1 still seems a good estimator at this lower limit of genome annotation.

**Table 3:** GO annotation statistics

**Dataset**	**% Products**	**Annotations**	**BP terms**
*S. cerevisiae*	78.0%	10622	2296
*C. albicans*	15.5%	1156	1116
*S. pombe* non-IEA	75.3%	8023	2267
*S. pombe* all.	80.5%	9981	2321

Statistics computed from the GO release 2007–12 corresponding to the following organisms: *S. cerevisiae*, *C. albicans *and *S. pombe *(both excluding and including 'Inferred from Electronic Annotations').

#### Inferred from Electronic Annotations (IEA)

Assignment of GO terms to gene products can be inferred from electronic annotations that have not yet been reviewed by a curator. Therefore, it might be desirable in some cases to rely only on expert-validated annotations. As all the annotations provided for *S. cerevisiae *are expert-validated (non-IEA codes), we analyzed GO cellular components for a closely similar organism for which IEA annotations are plentiful: *Schizosaccharomyces pombe *(*S. pombe*). Nearly 20% of the assignments of biological process (BP) terms were inferred from electronic annotations with 270 products annotated only with IEA codes. The electronic annotations increase the number of BP terms per product (from 2.0 to 2.4) and also increase the number of cellular components analyzed. The analyses performed with and without IEA annotations give very similar results (see Figure 3).

### Analysis of protein-protein network modules

Some recent work in the analysis of protein-protein interactions (PPIs) has concentrated on the detection of the modular organization of cellular function [35]. A functional module can be described as a group of physically or functionally linked molecules that work together to achieve a relatively distinct function [36]. Macromolecular complexes, cellular components and biological pathways are well-known examples of functional modules. Generally, computational methods try to find functional modules from a PPI network fulfilling topological constraints (e.g. densely connected regions for protein complexes), which are further tested for a common cellular function or relationship to an already-described complex. Nevertheless, there is a lack of reliable criteria for evaluating the quality of complexes derived from the analysis of PPI networks, making it difficult to assess the biological relevance of the derived modules [37].

Information about the overlap with known complexes, cellular co-localization, average semantic similarities for pairs of interacting proteins, and phenotype divergence [37-39] has been used to assess the quality of modules obtained from network analysis. As the preliminary results obtained from our study of MIPS complexes show (see Figure 2), there are proportionately more significant sets within the curated complexes than among the complexes obtained from systematic analysis [32-34]. This suggests that our method can be used to qualify a potential module in terms of its homogeneity and completeness through the analysis of 'biological process' annotations.

**Figure 4:**
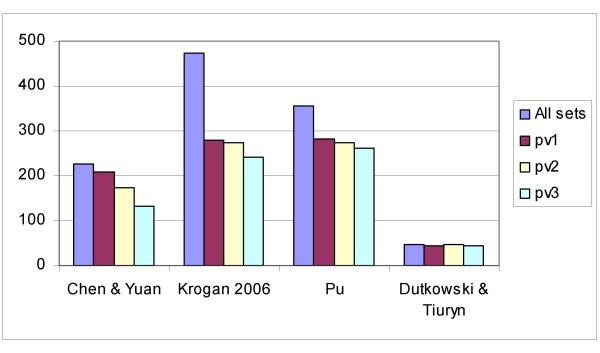
**Significant functional modules**. Total number of protein sets and significant sets (pv < 0.05) corresponding to the functional modules obtained from the analysis of protein-protein interaction networks: Chen & Yuan [39], Krogan *et al*. [38], Pu *et al*. [37], Dutkowski & Tiuryn [42].

Therefore, we computed the coherence score (and its statistical significance) of a series of functional modules obtained from more recent computational analyses of *S. cerevisiae *PPI networks (see Figure 4 for results). These datasets correspond to the following studies:

• Chen & Yuan [39] used an extension of a betweenness-based partition for analyzing a weighted graph built from the integration of various proteomics and microarray datasets.

• Krogan *et al*. [38] obtained a new TAP-MS interaction network and used a Markov clustering algorithm to detect complexes.

• Pu *et al*. [37] performed a comparative study of PPI networks, analyzing *inter alia *a Consolidated PPI network [40] that included data from Krogan *et al*. [38] and Gavin *et al*. [41].

• Dutkowski & Tiuryn [42] detected conserved functional modules through the alignment of yeast, worm and fly PPI networks. We have analyzed the protein sets corresponding to yeast proteins in these modules.

The conserved modules identified by Dutkowski & Tiuryn [42] show the highest percentage of significant sets, although they describe fewer modules. In their analysis, evolutionary constraints were used as a guarantee to ensure the biological significance of functional units.

Moreover, the proportion of significant complexes is greater in the data obtained by the analysis of the Consolidated network by Pu *et al*. [37] than in those obtained by Krogan *et al*. [38]. Therefore, this larger proportion of significant complexes agrees with other quality parameters computed by [37], namely overlap with known complexes and co-localization.

## Discussion

In this work we present a descriptive method, based on the analysis of functional annotations, for scoring the degree of homogeneity of a protein set and assessing its significance in the context of a reference set. The method has been evaluated using positive and randomly created datasets. Analysis of known biologically meaningful protein sets corresponding to macromolecular complexes, cellular components and pathways of *S. cerevisiae *revealed that most of them are significant in the context of the organism used as reference. However, the coherence scores obtained vary considerably, from very homogenous sets to fairly heterogeneous. This shows that the overall similarity of functional annotations (i.e. the coherence score) is not a good indicator of the functional completeness and separation of a protein set in the context of an organism. Therefore, in addition to measuring the functional homogeneity, a statistical assessment is performed.

The coherence score proposed in this work is based on previously-defined pairwise functional similarities. Pairwise similarity methods are increasingly used in quite different bioinformatics applications, such as prediction of protein-protein interaction data [43], priorization of disease candidate genes [44], missing value estimation in microarray data [45] and prediction of novel gene function [6]. Nevertheless, they have not so far been used to quantify the functional homogeneity of a protein set. For example, the average semantic similarity of interacting proteins was previously used by Pu *et al*. [37] to evaluate the quality of modules obtained from network analysis. The coherence score described in this work is expected to correlate with that measure, since it is defined as the average pairwise similarity between all distinct protein pairs. Nevertheless, the two measures are not directly comparable, for two reasons. First, the average similarity was obtained by Pu *et al*. for pairs of interacting proteins within the same module. In contrast, the coherence score in the present work is computed over all protein-protein pairs within a protein set, as we are not using data on interactions themselves. Secondly, pairwise similarity is computed using dissimilar approaches. The similarity used by Pu *et al*., as described in [21], is computed by averaging all functional term-term similarities between two proteins. Specifically, a similarity is first established among functional terms, using information from the GO hierarchy, and then similarity between proteins is computed by averaging pairwise term similarities. As semantic similarity accounts for the average term-term similarities of two proteins, it might underestimate or overestimate overall similarity, in contrast to the cosine and Jaccard similarities used in the present work, which exhibit a wider range of values, from 0 (no common terms) to 1 (exactly the same terms).

**Figure 5:**
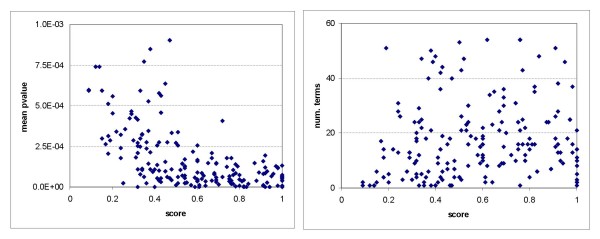
**Coherence score vs. enrichment-based metrics**. Coherence score versus metrics computed from the output of enrichment methods using 'biological process' annotations: (a) mean p-value of significant terms; (b) number of significant terms.

In addition to providing a coherence score, our method assesses the statistical significance of the set in the context of the global functional space of a reference, providing an additional quality parameter. This statistical assessment is performed globally, as all functional annotations of each protein in the set are considered together in the analysis. In contrast, most enrichment methods perform a local statistical assessment, as they analyze each functional term individually. In this way, they provide a collection of functional annotations together with their significance values. Therefore, they are mainly used by experts in order to support the functional interpretation of their experimental results. Nevertheless, it is not straightforward to use the information provided by enrichment methods to account for the functional homogeneity of a protein set. For instance, neither the number of enriched functional terms nor the averaged p-values of significant terms have previously been found to be good indicators of the homogeneity of a protein set [27]. In order to compare these enrichment-based measures with the coherence score proposed in this work, we analyzed the modules obtained by Pu *et al*. [37], using GO 'biological process' terms in both cases. Figure 5 shows the relationship between coherence score and averaged p-values of significant terms, as well as the number of enriched functional terms for those sets with at least three proteins annotated, where at least one term was found to be significant. Enrichment was performed using the cumulative hypergeometric distribution of each functional term annotated in the set (no correction was performed for multiple testing). As shown in Figure 5, and in agreement with previous studies using literature analysis [27], there is no clear correlation between the global coherence score and the two enrichment-based measures.

Therefore, the coherence score and corresponding p-values are shown to be valuable indicators of the global functional homogeneity of a protein set, complementing the functional analysis performed by currently available methods. To illustrate the type of information provided by our method and other functional methods, as well as their complementary relationship, we provide the results of the analysis of 'biological process' annotations of one of the functional modules obtained in [37]: 'Module 39' (see additional file 2). The exact application of the coherence score together with other functional analysis methods will depend on the type of analysis desired. If homogeneous sets are expected, our method can be used for validation in order to discard those that are heterogeneous. This is the case for the discovery of functional modules from protein-protein interaction networks, where protein clusters can first be filtered on the basis of functional homogeneity, avoiding any additional functional interpretation of those cases that are clearly heterogeneous. In contrast, if novel functional associations are sought, further analysis should be performed on those sets that are not highly homogeneous.

Both the coherence score and significance measures are computed from a set of functional annotations, from which as a first step a similarity is established. This similarity therefore depends, among other things, on the completeness of a genome annotation. In addition, we have applied our method to the analysis of *S. cerevisiae *sets, using an alternative similarity measure (Jaccard), to an incipient annotation project, *C. albicans*, and to a genome with nearly 20% of biological process term annotations inferred from electronic resources, *S. pombe*. As with other methods based on functional annotations, the completeness of annotations is by far the most important limiting factor in our methodology.

Finally, to illustrate the usefulness of our method, we have applied it to various protein sets corresponding to hypothetical functional modules and complexes obtained from PPI network analysis. Our results seem to agree with and complement other validation criteria, such as evolutionary conservation and overlap with known complexes.

## Conclusion

We have presented a method that scores the degree of homogeneity, or coherence, of a protein set on the basis of the global similarity of their functional annotations. It uses statistical hypothesis testing to assess the significance of the set in the context of the functional space of a reference set.

We can conclude that our method is complementary to previous descriptive functional analysis approaches. On the one hand, like enrichment methods, it analyzes a protein set. On the other, like some pairwise similarity methods, it measures the functional relatedness of proteins from a global point of view. Finally, as in enrichment methods, a statistical test is performed, in our case to evaluate the significance of the global coherence score of the protein set in the context of a reference set. However, in contrast to enrichment methods, it does not provide a functional interpretation of the protein set, as it reports two numerical values (coherence score and corresponding p-value) but not functional terms. As such it is a good filter prior to functional interpretation in cases where numerous protein sets are obtained (e.g. protein clusters obtained from protein interaction networks, gene expression clusters).

The coherence score and corresponding significance measures proposed in this work can be therefore used for validation of experimental sets where functionally homogeneous protein groups are expected. This is the case for – *inter alia *– cluster and bicluster analysis of gene expression profiles, protein-protein interaction clusters and sets of hypothetically homologous proteins.

## Authors' contributions

MC conceived the work, programmed the method, performed the analyses and wrote the manuscript. APM and JMC revised both the methodology and manuscript. All authors read and approved the final manuscript.

## Supplementary Material

Additional file 1 Coherence score and significance measures of random sets. Click here for file

Additional file 2 Functional analysis of 'Module 39' obtained by Pu *et al*. [37] using various approaches. Click here for file
