# Supplementary material for: Assessment of protein set coherence using functional annotations
Source: BMC Bioinformatics. 2008 Oct 20;9:444. doi: 10.1186/1471-2105-9-444 (PMC2588600; doi:10.1186/1471-2105-9-444)
Supplement: Additional file 1 — Coherence score and significance measures of random sets. [file 1471-2105-9-444-S1.pdf]

Chagoyen M, Carazo JM and Pascual-Montano A.

Assessment of protein set coherence using functional annotations

Supplementary data

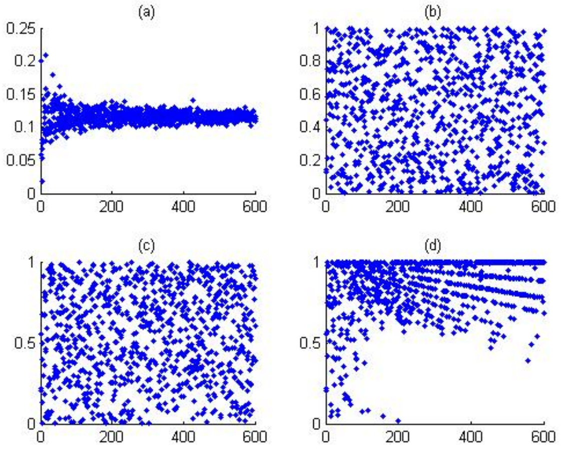

Figure. (a) Coherence score and significance measures (b) pv1, (c) pv2 and (d) pv3 of random sets, plotted against set size.
