# Supplementary material for: Assessment of protein set coherence using functional annotations
Source: BMC Bioinformatics. 2008 Oct 20;9:444. doi: 10.1186/1471-2105-9-444 (PMC2588600; doi:10.1186/1471-2105-9-444)
Supplement: Additional file 2 — Functional analysis of 'Module 39' obtained by Pu et al. [37] using various approaches. [file 1471-2105-9-444-S2.pdf]

Functional analysis of Module 39

Module 39 obtained by Pu *et al.* 2007 is comprised of 8 *S. cerevisiae* proteins:

Protein	Description	GO BP
SIC1p	Inhibitor of Cdc28-Clb kinase complexes that controls G1/S phase transition, preventing premature S phase and ensuring genomic integrity; phosphorylation targets Sic1p for SCF(CDC4)-dependent turnover; functional homolog of mammalian Kip1	* G1/S transition of mitotic cell cycle<br>* negative regulation of transposition, RNA-mediated<br>* regulation of cyclin-dependent protein kinase activity
CKS1p	Cyclin-dependent protein kinase regulatory subunit and adaptor; modulates proteolysis of M-phase targets through interactions with the proteasome; role in transcriptional regulation, recruiting proteasomal subunits to target gene promoters	* regulation of cell cycle<br>* transcription
CLN1p	G1 cyclin involved in regulation of the cell cycle; activates Cdc28p kinase to promote the G1 to S phase transition; late G1 specific expression depends on transcription factor complexes, MBF (Swi6p-Mbp1p) and SBF (Swi6p-Swi4p)	* regulation of cyclin-dependent protein kinase activity
CLB4p	B-type cyclin involved in cell cycle progression; activates Cdc28p to promote the G2/M transition; may be involved in DNA replication and spindle assembly; accumulates during S phase and G2, then targeted for ubiquitin-mediated degradation	* G2/M transition of mitotic cell cycle<br>* regulation of cyclin-dependent protein kinase activity<br>* S phase of mitotic cell cycle
CLN2p	G1 cyclin involved in regulation of the cell cycle; activates Cdc28p kinase to promote the G1 to S phase transition; late G1 specific expression depends on transcription factor complexes, MBF (Swi6p-Mbp1p) and SBF (Swi6p-Swi4p)	* negative regulation of transposition, RNA-mediated<br>* re-entry into mitotic cell cycle after pheromone arrest<br>* regulation of cyclin-dependent protein kinase activity
CDC28p	Catalytic subunit of the main cell cycle cyclin-dependent kinase (CDK); alternately associates with G1 cyclins (CLNs) and G2/M cyclins (CLBs) which direct the CDK to specific substrates	* negative regulation of meiotic cell cycle<br>* negative regulation of mitotic cell cycle<br>* negative regulation of transcription, DNA-dependent<br>* positive regulation of DNA replication during S phase<br>* positive regulation of meiotic cell cycle<br>* positive regulation of mitotic cell cycle<br>* positive regulation of transcription, DNA-dependent<br>* protein amino acid phosphorylation<br>* regulation of budding cell apical growth<br>* regulation of double-strand break repair via homologous recombination<br>* regulation of filamentous growth
CLB3p	B-type cyclin involved in cell cycle progression; activates Cdc28p to promote the G2/M transition; may be involved in DNA replication and spindle assembly; accumulates during S phase and G2, then targeted for ubiquitin-mediated degradation	* G2/M transition of mitotic cell cycle<br>* regulation of cyclin-dependent protein kinase activity<br>* S phase of mitotic cell cycle
SRL3p	Cytoplasmic protein that, when overexpressed, suppresses the lethality of a rad53 null mutation; potential Cdc28p substrate	* nucleobase, nucleoside, nucleotide and nucleic acid metabolic process

Information on proteins was obtained from the SGD (http://www.yeastgenome.org)

Analysis of enriched annotations:

Performed with SGD GO Term Finder. http://db.yeastgenome.org/cgi-bin/GO/goTermFinder.pl

Terms with P-value < 0.001 are shown in the following table.

GOID	GO_term	Cluster frequency	Background frequency	P-value	Gene(s) annotated to the term
79	regulation of cyclin-dependent protein kinase activity	5 out of 8 genes, 62.5%	19 out of 7158 background genes, 0.3%	1.74E-10	CLB3:SIC1:CLB4:CLN1:CLN2
51726	regulation of cell cycle	7 out of 8 genes, 87.5%	158 out of 7158 background genes, 2.2%	7.37E-10	CKS1:CDC28:CLB3:SIC1:CLB4:CLN1:CLN2
43549	regulation of kinase activity	5 out of 8 genes, 62.5%	31 out of 7158 background genes, 0.4%	2.53E-09	CLB3:SIC1:CLB4:CLN1:CLN2
45859	regulation of protein kinase activity	5 out of 8 genes, 62.5%	31 out of 7158 background genes, 0.4%	2.53E-09	CLB3:SIC1:CLB4:CLN1:CLN2
51338	regulation of transferase activity	5 out of 8 genes, 62.5%	32 out of 7158 background genes, 0.4%	3.00E-09	CLB3:SIC1:CLB4:CLN1:CLN2
50790	regulation of catalytic activity	5 out of 8 genes, 62.5%	61 out of 7158 background genes, 0.9%	8.77E-08	CLB3:SIC1:CLB4:CLN1:CLN2
65009	regulation of molecular function	5 out of 8 genes, 62.5%	65 out of 7158 background genes, 0.9%	1.21E-07	CLB3:SIC1:CLB4:CLN1:CLN2
50794	regulation of cellular process	7 out of 8 genes, 87.5%	891 out of 7158 background genes, 12.4%	0.00014	CKS1:CDC28:CLB3:SIC1:CLB4:CLN1:CLN2
50789	regulation of biological process	7 out of 8 genes, 87.5%	906 out of 7158 background genes, 12.7%	0.00015	CKS1:CDC28:CLB3:SIC1:CLB4:CLN1:CLN2
18	regulation of DNA recombination	3 out of 8 genes, 37.5%	51 out of 7158 background genes, 0.7%	0.00078	CDC28:SIC1:CLN2

Enrichment analysis provides a view on significant local functions. In this case:

- Most significant biological processes are ‘regulation of cyclin-dependent protein kinase activity’ (annotated in 5 proteins of the set) and ‘regulation of cell-cycle’ (annotated in 7 proteins).

Only a view on the hierarchy of GO terms (which is also provided in a graphical representation by SGD GO Term finder), allows us to check that

- ‘regulation of cyclin-dependent protein kinase activity’ is a child term of ‘regulation of cell-cycle’.
- Except ‘regulation of DNA recombination’, the rest of significant terms shown on the table are redundant (they are either parents of ‘regulation of CDK activity’ or ‘regulation of cell-cycle’).

Not significant terms discarded and are therefore usually not considered in functional interpretation of the set.

Analysis of pair-wise similarities:

Similarities between all pairs in the set computed using cosine similarity of weighted representations (see Methods for details) are:

SIC1	CSK1	CLN1	CLB4	CLN2	SRL3	CLB3	CDC38	
1.0	0.4283	0.8605	0.8571	0.5359	0.0430	0.8571	0.3479	**SIC1**
	1.0	0.4978	0.3940	0.3100	0.4426	0.3940	0.4093	**CSK1**
		1.0	0.7915	0.6228	0.0500	0.7915	0.2037	**CLN1**
			1.0	0.4930	0.0395	1.0000	0.4313	**CLB4**
				1.0	0.0311	0.4930	0.1459	**CLN2**
					1.0	0.0395	0.1811	**SRL3**
						1.0	0.4313	**CLB3**
							1.0	**CDC38**

This type of pair-wise similarity analysis provides a view on global function relationships (all terms are taken into consideration). The analysis is able to highlight, among others, that:

- CLB3p and CLB4p are in fact annotated with exactly the same terms (their similarity is equal to 1.0).
- Most closely related proteins from their overall annotations are SIC1p, CLN1p, CLB3p and CLB4p (with similarities higher than 0.85).
- Most dissimilar protein to other proteins in the set is SRL3.

Pair-wise similarities can be used to further perform other type of analysis like, e.g. functional clustering of the set, or analysis of coherence (see next).

Analysis of coherence:

Performed by the Method described in the manuscript (Chagoyen *et al.*)

GO aspect	Score	Size	p-value	s	r-s
Biological process	0.43	8	pv1: 8.51E-10<br>pv2: 7.42E-12<br>pv3: 8.05E-08	4<br>6<br>7	7<br>28<br>363
Cellular component	0.65	8	pv1: 5.42E-02<br>pv2: 2.99E-03<br>pv3: 1.41E-02	4<br>7<br>7	1086<br>1872<br>2378

Analysis of coherence provides a view on the global homogeneity and functional significance of the set:

- The protein set (module 39) has a coherence of 0.43 according to GO 'biological process' annotations.
- Although globally it is not a very homogeneous set, it is highly significant in the context of all proteins annotated in *S. cerevisiae* ('biological process' annotation different from unknown). E.g., according to pv1 there are 4 proteins within the set whose similarity to the set is higher than 0.43, while only 7 more in the reference fulfil this criterion.

If we perform a similar analysis using GO 'cellular component' annotations instead, the coherence of the set is 0.65. Nevertheless, in this case the set is not significant or fairly significant. E.g. according to pv1 (>0.05) as there are 4 proteins within the set whose similarity to the set is higher than 0.65, while there are 1086 more in the reference that also fulfil this criterion. If we consider pv2(<0.05) we find 7 proteins within the set whose similarity to the core is higher than 0.65, while there are 1872 other proteins in the reference that fulfil this criterion.
